# Society Meetings

**Published:** 1898-11

**Authors:** 


### SOCIETY MEETINGS.

THE Buffalo Academy of Medicine held meetings during the month of October, 1898, as follows :

*Section on Surgery.*—Tuesday evening, October 4th, program : On the use of chloroform in adenoid operations, Dr. F. W. Hinkel ; discussion opened by Drs. Renner and Mulford. Tertiary syphilis of the nose and pharynx, Dr. W. Scott Renner ; discussion by Drs. Hinkel, Wende and others. Report of case by Dr. J. Clemesha.

*Section on Medicine.*—Tuesday evening, October 11th, program : The Schott treatment in fatty degeneration of the heart, by Dr. Allen A. Jones. Remarks on prognosis and cause of death in cardiac diseases, by Dr. John H. Pryor. Serious heart disease without rheumatism, by Dr. A. L. Benedict.

*Section on Pathology.*—Tuesday evening, October 18th, program : Pathologic effect of devitalized teeth, by Dr. W. C. Barrett, M. D., D. D. S., dean of the dental department, University of Buffalo. Exhibitions of specimens.

*Section on Obstetrics.*—Wednesday evening, October 26th, program : The position of the Catholic church on obstetric practice, by Rev. J. F. Mooney. Purulent Ophthalmia, by Dr. Lucien Howe ; discussion by Drs. Hubbell, Starr and Satterlee.

---

THE Sanatory Club of Buffalo proposes to open its winter course with a discussion of the details of construction of hygienic camps of instruction—the permanent camps in which the great work of transforming the recruit into the soldier is done. The scope of the prospective discussion seems to indicate the conviction of the club that the prevention of disease in military camps waits upon a more complete use and application of well-known sanitary provisions—good drainage and pure water, whereby we secure clean soil, pure air and healthful diet. The enterprise of the club in this direction is to be commended, and it is earnestly hoped that a full attendance of members as well as a large audience of citizens generally will listen to the discussion.

---

The Medical Society of the State of New York will hold its ninety-third annual meeting at Albany, Tuesday, Wednesday and Thursday, January 31 and February 1 and 2, 1899, under the presidency of Dr. John O. Roe, of Rochester. The business committee has just been announced and is as follows: Dr. Willis G. Macdonald, Albany, chairman; Dr. Edward B. Angell, Rochester; Dr. James M. Winfield, Brooklyn. Communications relating to the program should be addressed to one of these gentlemen.

The Mississippi Valley Medical Association at its recent meeting at Nashville, Tenn., elected the following-named officers: President, Dr. Duncan Eve, Nashville, Tenn.; first vice-president, Dr. A. J. Ochsner, Chicago, Ill.; second vice-president, Dr. J. C. Morfit, St. Louis, Mo.; secretary, Dr. Henry E. Tuley, Louisville, Ky. (111 W. Ky Street); treasurer, Dr. Dudley S. Reynolds, Louisville, Ky. Chicago is to be the next place of meeting. Chairman of committee of arrangements, Dr. Harold N. Moyer. Time of meeting: October, 1899. Date to be determined by the executive officers and the chairman of the committee of arrangements.

The Southern Surgical and Gynecological Association previously announced its eleventh annual meeting to be held in Memphis, Tenn., Tuesday, Wednesday and Thursday, November 8, 9 and 10, 1898, but it has been postponed till Tuesday, Wednesday and Thursday, December 6, 7 and 8, 1898, on account of the quarantine regulations relating to yellow fever in some parts of the South. The Gayoso House has been selected as headquarters for the Association. Dr. Richard Douglas, of Nashville, is president and Dr. Wm. E. B. Davis, of Birmingham, is secretary.

The Western Surgical and Gynecological Association will hold its eighth annual meeting at Omaha, December 28 and 29, 1898, under the presidency of Dr. D. S. Fairchild, of Clinton, Ia. Titles of papers from some of the leading surgeons of the west are already in the hands of the secretary and the coming meeting promises to be the most interesting yet held. The local committee of arrangements at Omaha is actively preparing for the entertainment and comfort of those who attend. Titles of papers should be sent to the secretary, Dr. Geo. H. Simmons, Lincoln, Neb., not later than November 20th to insure a place on the program.

---

The Esculapian Club is the name of a new medical organisation that has been established in Buffalo. It holds meetings every month from October to May inclusive and its membership is limited to twenty-five. The November meeting will be held at the residence of Dr. C. J. Reynolds, 322 Fox street, at which Dr. A. W. Bayliss will read a paper. A notice of this medical society would have been incorporated in the Century of Medical History, now running in the Journal, had the historian been advised of its existence.

---

The Iowa State Association of Railway Surgeons held its fifth annual meeting at Clinton, October 13 and 14, 1898, under the presidency of Dr. R. A. Patchen, of Des Moines. The program was elaborate and the meeting proved excellent. Dr. Patchen is a native of Western New York and on a recent visit East paid the Journal a call, which courtesy we shall be glad to have often repeated.

